# Comparison of Progression Rate of Retinal Pigment Epithelium Loss in Patients with Neovascular Age-Related Macular Degeneration Treated with Ranibizumab and Aflibercept

**DOI:** 10.1155/2017/7432739

**Published:** 2017-02-20

**Authors:** Juliana Wons, Magdalena A. Wirth, Nicole Graf, Matthias D. Becker, Stephan Michels

**Affiliations:** ^1^City Hospital Triemli, Zurich, Switzerland; ^2^Graf Biostatistics, Winterthur, Switzerland; ^3^University of Zurich, Zurich, Switzerland

## Abstract

*Purpose.* Retinal pigment epithelium (RPE) loss in neovascular age-related macular degeneration (nAMD) seem to have a linear progression but might be influenced by the treatment. The purpose of the study is the comparison of RPE loss over three years in patients treated with intravitreal ranibizumab to patients who were switched to aflibercept. *Methods.* A retrospective analysis with 96 eyes switched to aflibercept was conducted. The progression rate of RPE loss was evaluated in patients who showed atrophy one year prior to switch (*n* = 17) or on switch date (*n* = 19). The RPE loss was evaluated by spectral domain optical coherence tomography (SD-OCT). Further, 22 eyes from patients treated with ranibizumab were compared. *Results.* The median yearly progression of RPE loss after square root transformation showed no significant difference in the year prior to switch compared to the year after switch (*p* = 0.854). In patients who received only ranibizumab, the median yearly progression of RPE loss was 0.15 mm/y, for aflibercept patients, 0.13 mm/y. This difference was not statistically significant (*p* = 0.172). *Conclusions.* There seems to be a linear progression rate of RPE loss in patients treated with ranibizumab as well as in patients with aflibercept. No significant increase of progression rate was found after switch to aflibercept.

## 1. Introduction

Age-related macular degeneration (AMD) is a common cause for legal blindness in the elderly population reaching a global prevalence—according a recent meta-analysis of 129,664 individuals aged 45–85 years—of 8.69% [1].

Central vision is commonly impaired in AMD patients due to choroidal neovascularization (CNV) or due to fibrosis and/or loss of retinal pigment epithelium (RPE). CNV and associated edema can be controlled today by chronic treatment with inhibitors of the vascular endothelial growth factor (VEGF); however, fibrosis or loss of the RPE—once they occur—appears irreversible. Since fibrosis and RPE loss are associated with photoreceptor loss, central vision is impaired. RPE loss is common in nonneovascular AMD, but can also be associated with neovascular AMD (nAMD), either as sequelae of CNV or as concomitant nonneovascular AMD. Changes in the cell environment as a result of oxidative stress have been proposed to further damage RPE cells, which are already dysfunctional due to the underlying disease [2, 3].

Inhibition of VEGF is the current first-line therapy for nAMD and beneficial for most patients. However, VEGF is also a relevant physiologic factor within the retina and the RPE and its full suppression might impair the RPE and the underlying choriocapillaris [2, 4]. In the large Comparison of Age-Related Macular Degeneration Treatments Trials (CATT), more RPE atrophy appearance was found in patients treated on a monthly basis than those with pro re nata regiment with fewer treatments [5].

In addition to treatment frequency, different VEGF-binding affinities as well as other properties of currently used VEGF inhibitors might play a role on their effect on RPE and choroid. In effect, differences—especially on the choroid—have been described for ranibizumab and aflibercept in animal models and in clinical studies [4, 6].

In the past, fundus photography and fluorescein angiography (FA) have been the standard for evaluating RPE loss; however, there are key disadvantages in using these imaging modalities. Currently, fundus autofluorescence and SD-OCT have become the most widely used imaging technologies for evaluating loss of the RPE. Recently, it has been shown that RPE loss progression rates in nonneovascular AMD—after square root transformation—have a linear progression using different imagining modalities [7]. Despite the current advances in RPE imaging and potentially first upcoming treatment options to prevent RPE loss, there is no formal consensus terminology of RPE loss in AMD and its precise definition. One aspect however—most experts agree upon—is the total loss of RPE in areas with hypertransmission seen in SD-OCT.

In this context, a retrospective analysis on the progression rate of RPE loss over time in patients with nAMD being switched from ranibizumab or bevacizumab to aflibercept was conducted and compared to nAMD patients of similar age being treated with ranibizumab only.

## 2. Material and Methods

The retrospective study was conducted at the Department of Ophthalmology at the City Hospital Triemli in Zurich and included 118 eyes with nAMD. The study was conducted according to the Declaration of Helsinki and was approved by the local ethics committee in Zurich. Written informed consent was collected from all study subjects, prior to investigation-related procedures.

The study was conducted based on the following patient selection. A total of 96 eyes initially treated with ranibizumab or bevacizumab were switched to aflibercept due to insufficient response defined as persistent intraretinal or subretinal fluid despite at least three intravitreal injections within four months prior to the switch (consecutive cases between November 2012 and March 2013). Patients aged from 68 to 95 years were included in the study. For the statistical analysis, RPE loss lesions were included under the following conditions: RPE loss diameter was more than 300 *μ*m; atrophy lesions without signs of suspected CNV in SD-OCT including PED or fibrosis, entitled as CNV independent RPE loss; and RPE loss area fully within the 20° × 15° scan frame at one year before switch or on switch date.

All patients were imaged at all visits using the Heidelberg Spectralis system (Heidelberg Engineering, Heidelberg, Germany) in follow-up mode. A standard set of 19 B-scans (512 A-scans; 20° × 15°) was used at all times. The total area of hypertransmission on SD-OCT within the 20° × 15° and the maximum diameter of RPE loss on the fovea crossing OCT scans was measured by two graders (JW and MW).

Measurements were taken one year prior to switch, at the time of switch, and one, two, and in some patients as well three years after switch. The identical OCT setting was used for the follow-up scans. For independence of growth rate from initial lesion size a square root transformation was performed [8]. All eyes were treated, except for the loading dose at switch, using a treat-and-extend strategy as described recently [9].

In addition, the progression rate of RPE loss (hypertransmission on SD-OCT) was retrospectively examined in 22 patients with nAMD, who were treated only with ranibizumab over 3 years. Patients who developed RPE atrophy according to the same criteria as above within 4 to 40 months after initiation of ranibizumab therapy were included.

The yearly progression rates of RPE loss were statistically compared.

### 2.1. Image Analysis

**Figure 1:**
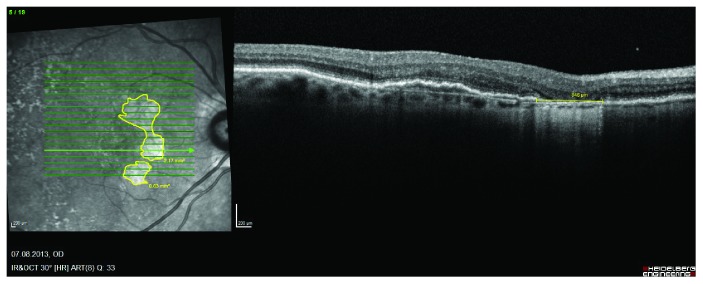
Representative case with RPE loss of a patient receiving intravitreal ranibizumab.

**Figure 2:**
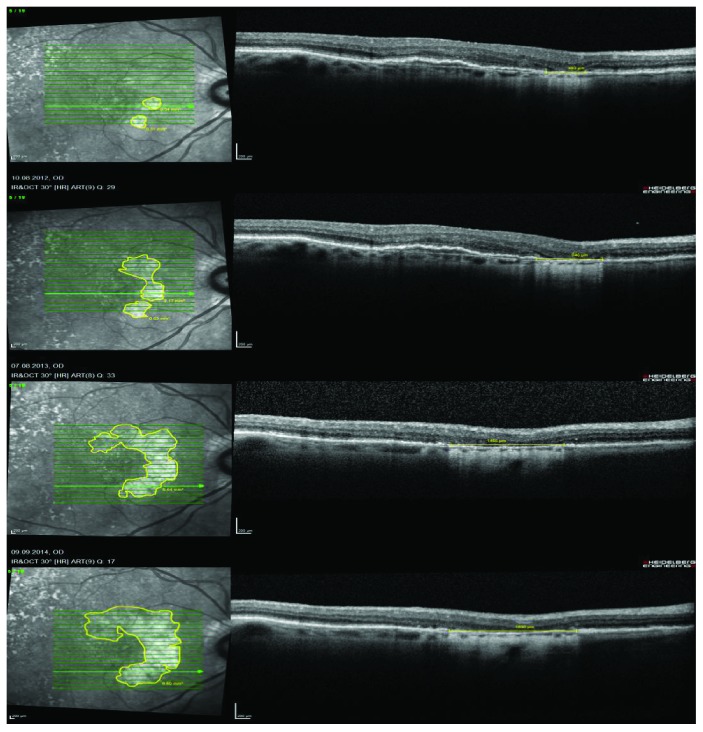
Yearly progression of RPE loss in the right eye of a patient with nAMD and intravitreal ranibizumab therapy.

The scanning laser image analysis program of the Heidelberg Eye Explorer (Version 1.9.10.0) was used to measure RPE loss. Total loss of RPE was predefined in the OCT B-scans by the pattern of increased choroidal hyperreflectivity due to hypertransmission and absence of the photoreceptor inner segment/outer segment junction (Figure 1). The total area was measured with the draw region tool—based on the OCT B-scans—in the infrared image. In addition, the maximum diameter of the absolute RPE loss was measured on the fovea crossing B-scan. The yearly progression rates—based on the square root transformation—were calculated (Figure 2).

### 2.2. Statistical Analysis

All analyses were conducted using SPSS Version 20. Figures were created in Graphpad Prism Version 5. For quantitative variables, mean, standard deviation (SD), median, 1st quartile (Q1), 3rd quartile (Q3), minimum (Min), and maximum (Max) are given.

All measurements of the area of RPE loss were square root transformed to account for different baseline values [8]. For comparison of RPE loss one year prior to and one year after switch to aflibercept, differences of square root transformed values were compared with an exact Wilcoxon signed rank test. For comparison of RPE loss between the treatment groups, curve estimation regression statistics was performed and the slopes of the square root transformed values were compared with an exact Mann-Whitney *U* test.

In addition, the injection rates of both treatment groups were calculated and compared with an exact Mann-Whitney *U* test or an exact Wilcoxon signed rank test for inter- and intragroup comparisons, respectively.

## 3. Results

### 3.1. Clinical Demographics

The study analyzed retrospectively 96 eyes of patients who were switched to aflibercept from ranibizumab or bevacizumab. The CNV independent lesions of RPE loss were measured. On each patient, the atrophy measurements were performed in only one eye.

All 96 eyes were retrospectively evaluated for hypertransmission on SD-OCT one year prior to switch regarding the above-mentioned inclusion criteria for RPE loss regions. A CNV independent RPE loss was present in 17 patients one year before switch. In 19 patients, a RPE loss could be measured on switch date. In 9 of these 19 patients, a follow-up exam of two years was possible; in 6 of these patients, a follow-up exam three years after switch was conducted.

The median age of the patients within the therapy switch group that was statistically analyzed was 85.16 years (SD ± 11.31). Ten of the treated eyes were from female patients.

Most of 96 patients were treated only with ranibizumab before switch. Regarding the 19 patients who were included into the statistical analysis for comparison between two treatment groups, two patients were treated with ranibizumab and bevacizumab before switch.

A total of 17 patients who showed RPE loss one year prior to switch could be included for an intraindividual comparison of RPE loss prior to and after switch to aflibercept. The median number of injections for these 17 patients 24 months before switch was 20 and 17 in a follow-up time of 24 months after switch. The injection rate over 24 months was in median 2 injections less per patient after switch; the difference was not statistically significant (*p* = 0.132).

**Figure 3:**
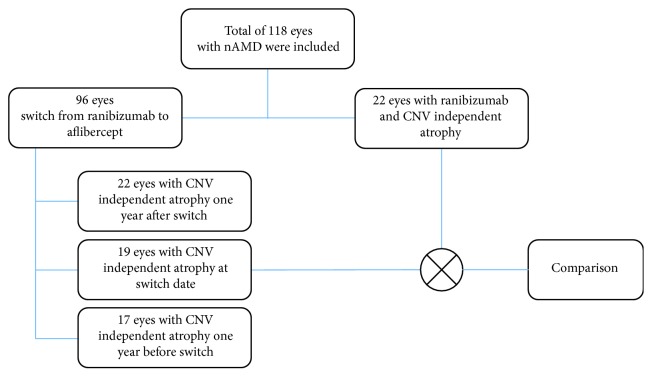
Overview of the included eyes and the different patient groups.

For a comparison between the two treatments, 22 patients with only ranibizumab therapy were compared to 19 patients after switch to aflibercept (Figure 3).

The median age of the ranibizumab patients was 87.36 years (SD ± 0.71). The mean injection rate of the ranibizumab eyes was 0.54 injections per month and 0.71 injections per month for the 19 aflibercept eyes. This difference was statistically significant (*p* = 0.011).

Details on the anti-VEGF treatments are shown in Tables 1 and 2.

The analysis included patients who showed nAMD with classic or occult CNV (analog to CNV lesion types 1 and 2) [10]. The examined RPE loss regions showed different patterns comparable to the aspects known from geographic atrophy in nonneovascular AMD (focal, diffuse, patchy, and banded) [11].

The intraclass correlation coefficient (ICC) of the two independent graders amounted to 0.98 (ICC 2.1).

### 3.2. Comparison of RPE Loss prior to and after Switch to Aflibercept

The median progression rate of RPE loss was after square root transformation with 0.11 mm identical in the year prior to and after switch to aflibercept (*p* = 0.854). Descriptive statistics for enlargement rate before and after switch to aflibercept is shown in Table 3.

**Figure 4:**
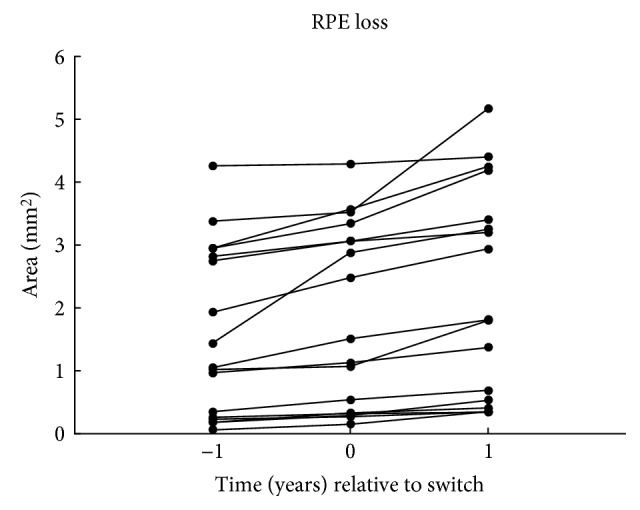
Course of RPE loss (*n* = 17).

The course of atrophy area over time before and after therapy switch is represented by Figure 4.

### 3.3. Comparison of Course of RPE Loss between Aflibercept and Ranibizumab Patients

The patients of comparable age treated with aflibercept or ranibizumab had no significant differences in the yearly RPE loss (after square root transformation). Table 4 shows the RPE baseline area of RPE loss for each drug.

**Figure 5:**
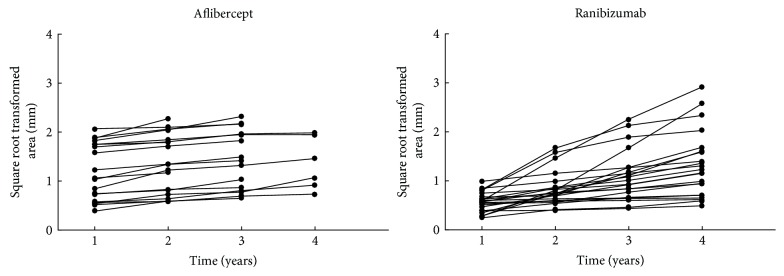
Course of RPE loss for patients receiving aflibercept (*n* = 19) and ranibizumab (*n* = 22).

Also within each group no significant change in the yearly rate of RPE loss could be found. The overall median yearly progression was 0.13 mm for aflibercept patients and 0.15 mm for ranibizumab patients (Table 5). This difference was not statistically significant (*p* = 0.172). The significant difference in the area of RPE loss at baseline was accounted for by using the square root transformation. Subsequently, the slope of the square root transformed area was calculated. *R*^2^ was between 0.858 and 1.000 with a mean of 0.959. Thus, all courses of RPE loss could be described as a linear regression (Figure 5).

Further details on each group (number of injections, size of RPE loss at baseline, and the RPE loss for each year and each drug) are shown in Tables 1 and 2.

## 4. Discussion

The presented study seems to show a linear progression of CNV independent RPE loss in patients with nAMD under anti-VEGF treatment over at least three years of treatment.

Our mean progression rate before switch is 0.30 mm^2^/year and 0.39 mm^2^/year after switch, which is similar to the average rate of progression in the work of Bhisitkul et al. [12]. However, we did not include CNV lesions in the atrophy measurements. Until now, it is not known if we can compare atrophy lesions in nAMD and nonneovascular AMD. Based on the limited data in the literature, we assume that atrophy progression could have similar characteristics, especially regarding the CNV independent RPE loss in nAMD.

The primary aim for our study was to investigate, whether there is an early detectable, significant difference in the growth of atrophy independent from the CNV region after anti-VEGF therapy switch. No significant acceleration or deceleration of RPE loss was found when patients were switched from ranibizumab or bevacizumab to aflibercept. The number of aflibercept injections in the year after switch was 1.6 injections lower than in the year prior to switch using ranibizumab. Fewer injections with aflibercept might have despite longer anti-VEGF effects in the eye [13] led to the same rate of RPE loss as a more frequently applied drug with shorter activity in the eye. However, in both groups being treated on one drug for several years with decreasing numbers of injections per year (Tables 1 and 2), RPE loss remained constant and no deceleration could be found.

This study might indicate that progression of CNV independent RPE loss in nAMD has a linear progression over time independent of the duration of treatment and the anti-VEGF drug used.

It has however to be taken into consideration that the selected population was initially insufficiently responding to anti-VEGF therapy with ranibizumab and bevacizumab. This selected patient population required quite extensive treatment prior to switch (mean of 18.5 injections/24 months) and was still frequently treated after switch to aflibercept (mean of 14.9 injections/24 months). This is on the one hand reassuring since—despite heavy treatment—no acceleration of RPE loss could be found over several years; however, these eyes might have high levels of endogenous produced VEGF potentially protective from RPE damage.

Our findings for yearly growth rates of atrophy regions are in line with previous studies for nAMD and for slow-growing atrophy in nonneovascular AMD [3, 11]. This might relate to a reduced number of anti-VEGF injections used in a treat-and-extend setting compared to monthly treatment. Based on clinical experience, intravitreal anti-VEGF injections can induce a reduction in choroidal thickness [14], which has been confirmed by in vivo studies in primates. Understandably the reduction in choroidal flow, a reduced fenestration in the choriocapillaris and a reduced choriocapillaris density—as shown in primates—could lead to impairment of the RPE [4]. We have shown priorly in our study population that intravitreal anti-VEGF therapy leads to a significant reduction in choroidal thickness and a switch to three monthly injections of aflibercept from prior intensive anti-VEGF therapy induces a further reduction in choroidal thickness [6]. Whether the choroidal thickness plays a significant role in the development of atrophy in nAMD is currently unclear. But there are study results for nonneovascular AMD that indicate a choroidal thinning in the eyes with geographic atrophy compared to normal eyes of similar age [15].

The treat-and-extend treatment strategy used in the study has an OCT-based proactive component; however, it is not a continuous VEGF suppression. Between injections, this treatment allows some recovery of VEGF levels, which might be sufficient to prevent accelerated RPE loss. Furthermore—even though intriguing—it has so far not been shown in a clinic that any reduction in choroidal thickness is necessarily associated with a RPE impairment.

Even though our study does not allow drawing absolute conclusion due to the absence of a today impossible control group (untreated nAMD), we could not detect a very different rate of RPE loss progression between anti-VEGF drugs with different affinities to VEGF as shown within the switching group and the group treated with ranibizumab only.

Limitations of the presented study are its retrospective nature, the limited number of patients showing any RPE loss one year after switch to aflibercept, and the relatively small sample size in general. Further, the limitation of RPE loss analysis based on SD-OCT with 19 B-scans and infrared imaging has to be mentioned.

Reassuring are the consistent RPE loss rates in long-term follow-up of several years in an identical follow-up imaging mode with minimal loss of patients in follow-up. Currently, there is no consistent terminology for RPE loss. Much of the terminology dates to the era of fundus photography and fluorescein angiography. We opted for OCT technology since image acquisition is easy and provides good quality even without mydriasis. In contrast, autofluorescence imaging shows much worse quality in undilated eyes and is quite uncomfortable for patients. To date there is no consensus on terminology using OCT technology for RPE imaging; however, hypertransmission associated with loss of the overlaying IS/OS band appears to be a consistent indicator for absolute RPE loss. This excludes however possibly impaired RPE or a reduced RPE density. It has been shown that different imaging modalities measure RPE loss differently; the progression rates in different modalities have however been quite consistent [3, 8].

It has to be mentioned that there could be an influence of different baseline RPE losses for the comparison of the two groups. As seen in Figure 5, some patients of the solely ranibizumab group had a stronger RPE loss increase. However, in this group also, the injection rates were higher as in comparison to the aflibercept patients after switch. The results of CATT showed more often development of atrophy in patients treated on a monthly basis than with pro re nata regiment with fewer treatments [5]. Maybe the increased RPE loss in this group can be a sign for the presumed argument that a higher number of injections lead to a faster RPE loss.

## 5. Conclusion

In conclusion, our data did not show an increase of CNV independent atrophy progression rate in nAMD after switch from bevacizumab or ranibizumab to aflibercept in comparison of follow-up exams from one year before until up to three years after switch. Further, a comparable RPE loss progression could be seen in a control group of patients, who received only ranibizumab over a three-year period.

## Figures and Tables

**Table 1:** Baseline characteristics of enrolled therapy switch patients.

Patients	RPE loss (mm^2^)					Number of injections (*N*)		
	One year before switch	On switch date	One year after switch	Two years after switch	Three years after switch	Total	Prior to switch 24 months	After switch 24 months
P1	0.18	0.29	0.53	0.60	1.15	*56*	13	22
P2	0.23	0.27	0.35	0.42		*53*	22	21
P3	0.18	0.33	0.41	0.65	0.84	*44*	20	24
P4	1.44	2.88	3.25	3.85	3.94	*28*	19	4
P5	1.05	1.51	1.81	2.23		*47*	22	18
P6	0.35	0.54	0.69	0.75		*30*	16	17
P7	2.95	3.34	4.19	5.37		*64*	20	18
P8	2.82	3.06	3.40	3.80	3.79	*53*	12	17
P9	4.26	4.29	4.40	4.68		*26*	13	13
P10	1.02	1.07	1.80	2.01		*39*	16	23
P11	0.26	0.32	0.34	0.48	0.54	*40*	21	16
P12	0.97	1.13	1.37			*61*	22	12
P13	2.95	3.57	4.25	4.72		*50*	20	18
P14	1.93	2.48	2.94	3.34		*44*	21	11
P15	2.75	3.06	3.20			*36*	14	6
P16	3.38	3.52	5.17			*46*	23	7
P17	0.06	0.15	0.35			*28*	20	7
P18	0	0.55	0.66	1.06		*9*	5	4
P19	0	0.71	1.50	1.74	2.13	*36*	19	17

**Table 2:** Baseline characteristics of enrolled ranibizumab patients.

Patients	RPE loss (mm^2^)				Number of injections (*N*)	
	First year	Second year	Third year	Fourth year	Total	Since RPE loss, 36 months
P1	0.29	0.31	0.59	0.90	*29*	18
P2	0.31	0.34	0.36	0.38	*47*	20
P3	0.06	0.17	0.21	0.35	*20*	6
P4	0.67	2.78	4.54	5.45	*6*	3
P5	0.08	0.54	1.62	2.84	*21*	16
P6	0.15	0.29	0.44	0.50	*31*	21
P7	0.45	0.48	0.85	1.33	*7*	2
P8	0.65	2.49	3.58	4.11	*26*	18
P9	0.40	0.75	1.17	1.85	*37*	26
P10	0.12	0.55	1.23	2.58	*35*	18
P11	0.39	0.55	0.70	0.88	*30*	23
P12	0.36	0.40	0.42	0.50	*31*	22
P13	0.08	0.65	2.80	6.64	*44*	22
P14	0.32	0.72	1.02	1.52	*27*	19
P15	0.26	0.36	0.41	0.42	*49*	19
P16	0.72	0.98	1.33	1.71	*27*	23
P17	0.33	0.58	0.70	0.98	*25*	20
P18	0.22	0.49	1.38	2.53	*22*	12
P19	0.56	0.68	0.86	1.35	*25*	21
P20	0.35	2.14	5.10	8.51	*18*	15
P21	0.15	0.16	0.19	0.24	*27*	18
P22	0.98	1.33	1.61	1.96	*39*	31

**Table 3:** Enlargement rate (mm) (*n* = 17).

Time	Mean	SD	Median	Q1	Q3	Min	Max
Prior to switch	0.12	0.11	0.11	0.05	0.16	0.01	0.50
After switch	0.14	0.10	0.11	0.07	0.20	0.02	0.40

**Table 4:** Baseline area of RPE loss.

Group	Mean	SD	Median	Q1	Q3	Min	Max
Aflibercept (*n* = 19)	1.74	1.42	1.13	0.33	3.06	0.15	4.29
Ranibizumab (*n* = 22)	0.36	0.24	0.33	0.15	0.48	0.06	0.98

**Table 5:** Relative increase of RPE loss.

Group	Mean	SD	Median	Q1	Q3	Min	Max
Aflibercept (*n* = 19)	0.14	0.09	0.13	0.07	0.19	0.04	0.40
Ranibizumab (*n* = 22)	0.25	0.22	0.15	0.11	0.40	0.02	0.78
